# Analysing Point Mutations in Protein Cleavage Sites by Using Enzyme Specificity Matrices

**DOI:** 10.3389/fendo.2019.00267

**Published:** 2019-05-03

**Authors:** Jakob Triebel, Sandeep Silawal, Maximilian Willauschus, Gundula Schulze-Tanzil, Thomas Bertsch

**Affiliations:** ^1^Institute for Clinical Chemistry, Laboratory Medicine and Transfusion Medicine, Nuremberg General Hospital and Paracelsus Medical University, Nuremberg, Germany; ^2^Department of Anatomy, Paracelsus Medical University, Nuremberg, Germany

**Keywords:** MEROPS, Ensembl, UniProt, MEGA, cleavage sites, enzymes, point mutations

Public databases contain a rapidly expanding amount of information about protein sequences of diverse species, genetic mutations in humans, as well as enzymes and their substrates. This opens new possibilities of data analysis, hypothesis generation, and testing. It is the purpose of the present article to briefly highlight two exemplary analyses which are of broad applicability: the effect of point mutations on the generation of hormones, and the investigation of the molecular evolution of hormonal systems.

The pituitary hormone prolactin, for example, can undergo proteolytic cleavage by cathepsin D and other proteases. Such cleavage can result in the generation of vasoinhibin, a hormone with a range of effects and signaling mechanisms distinct from uncleaved prolactin. It was projected that missense point mutations in human prolactin, which lead to an amino acid change within a specific cleavage site, can inhibit or facilitate the enzymatic cleavage, thus altering the levels of vasoinhibin (1). The general principle underlying this projection, is that point mutations in cleavage sites can affect the levels of the effector hormone which is arising from the cleavage of its precursor. This alteration of hormone levels may be clinically relevant in terms of a protective or an aggravating factor, depending on the function of the hormone or protein in a disease. Indeed, if leucine on position P1 (the first position next to the cleavage site NH2-terminal) is affected by a missense mutation and replaced by tryptophan, it is very likely that the cleavage that usually occurs at this site is significantly inhibited. This is because leucine on position P1 is the most preferred amino acid by cathepsin D, as shown by its entry in the MEROPS data base^1^ (2), which lists 416 observed cleavages in which leucine was present at this position in the substrate. For tryptophan, this number is merely 29, indicating a lower preference of cathepsin D for this amino acid at this position. If, in another scenario, serine in P1 (12 observed cleavages) is affected by a mutation and replaced by leucine, it is likely that the change significantly enhances the proteolytic cleavage, because, as mentioned, leucine is the most preferred amino acid at P1. Of note, there is experimental evidence supporting this type of projection as single amino acid substitutions in prolactin and the subsequent cleavage efficiency tested with cathepsin D correspond well with the expected changes according to the numbers of observed cleavages in the MEROPS enzyme specificity matrix (1, 3).

When analyzing point mutations in prolactin located in cleavage sites generating vasoinhibin, retrieved from the Ensembl-database^2^ (4), significant changes of the projected cleavage efficiency were found. The cleavage efficiency was determined by addition of the numbers of observed cleavages for each amino acid at a particular position, generating an 8P-score as a surrogate parameter for the efficiency of a given sequence. For example, the mutation leading to the replacement of methionine by valine in position P3 of a cleavage site for the generation of a 15 kDa vasoinhibin-isoform, resulted in an increase of the 8P-score by 68 points, suggestive of a higher cleavage efficiency because of this point mutation (1) (Table 1). In the case of an alteration of vasoinhibin levels because of enhanced or inhibited cleavage, the clinical relevance of the point mutations can be assumed for diseases in which a dysregulation of vasoinhibin levels has been reported, such as diabetic retinopathy (5) and peripartum cardiomyopathy (6). Point mutations in cleavage sites may therefore constitute a “second-hit”-factor in the pathophysiology of diseases in which the cleavage product is an important detrimental player, or they could constitute a protective factor. Hence, we propose that this type of analyses of detected point mutations for which no phenotype is known, will lead to the identification of clinically relevant point mutations. As a matter of course, the clinical relevance of mutations can only be demonstrated if these mutations are found in patients with the disease under investigation and if their effect on the cleavage is experimentally confirmed, but the theoretical projection of the cleavage efficiency will help to identify promising candidates. On the background of the many hormones and other proteins which arise from enzymatic cleavage of a precursor, for example adrenocorticotropic hormone and insulin, this appears as a rewarding option.

**Table 1:** Effect of mutation p.M158V in prolactin on cleavage efficiency.

**Cleavage site for the 15 kDa vasoinhibin isoform in prolactin generated by Cathepsin D, ancestral sequence**								
Amino acid position	157	158	159	160	161	162	163	164
Cleavage site position	P4	P3	P2	P1	P1′	P2′	P3′	P4′
Amino acid	Gly	Met	Glu	Leu	Ile	Val	Ser	Gln
Merops specificity CD	42	26	94	416	119	131	55	52
8P-score	935							
**Prolactin sequence carrying point mutation p.M158V, ID: rs749978269**								
Amino acid position	157	158	159	160	161	162	163	164
Cleavage site position	P4	P3	P2	P1	P1'	P2'	P3'	P4'
Amino acid	Gly	Val	Glu	Leu	Ile	Val	Ser	Gln
Merops specificity CD	42	94	94	416	119	131	55	52
8P-score	1003 (+68)							

*The point mutation rs749978269 leading to a substitution of methionine by valine in position 158 is projected to increase the cleavage efficiency of cathepsin D (CD) at this site, according to the MEROPS specificity data for cathepsin D and the calculated 8P-score. Modified from Table 1 as published by Triebel et al. (1)*.

Another application of the strategy using the enzyme substrate specificity data from MEROPS is its use in conjunction with protein sequences retrievable from UniProt^3^ (7) or Ensembl. Multiple protein sequence alignments of sequences from various species can be build, using MEGA^4^ (8) for example, and the conservation of cleavage sites can be evaluated, including an estimation of how the cleavage changes in response to sequence variations. This will likely produce valuable insights into the evolution of endocrine systems during speciation, but also into the evolution of any other protein which is generated by posttranslational proteolytic processing.

The use of MEROPS and its possible applications has been described in detail by its creators, Rawlings et al. (2). Here, we briefly point out methods which can be used to identify clinically relevant point mutations and to generate insights into the evolution of hormones and other proteins which emerge from proteolytic cleavage of a precursor. This shows once more the great value that emerges from public databases such as MEROPS, UniProt, and Ensembl. The existence of these databases, the efforts of their creators, curators, and funding bodies are much appreciated by the authors.

## Author Contributions

JT wrote the manuscript. SS, MW, GS-T, and TB edited the manuscript. All authors approved the final version.

### Conflict of Interest Statement

The authors declare that the research was conducted in the absence of any commercial or financial relationships that could be construed as a potential conflict of interest.

## Footnotes

^1^Available online at: https://www.ebi.ac.uk/merops/

^2^Available online at: http://www.ensembl.org

^3^Available online at: https://www.uniprot.org/

^4^Available online at: https://megasoftware.net/
